# Role of noncoding RNAs in orthodontic tooth movement: new insights into periodontium remodeling

**DOI:** 10.1186/s12967-023-03951-9

**Published:** 2023-02-09

**Authors:** Yuming Chen, Chao Zhang

**Affiliations:** Stomatological Hospital, Southern Medical University, Guangzhou, 510280 China

**Keywords:** Orthodontic tooth movement, Noncoding RNA, Periodontium remodeling, Orthodontic treatment

## Abstract

Orthodontic tooth movement (OTM) is biologically based on the spatiotemporal remodeling process in periodontium, the mechanisms of which remain obscure. Noncoding RNAs (ncRNAs), especially microRNAs and long noncoding RNAs, play a pivotal role in maintaining periodontal homeostasis at the transcriptional, post-transcriptional, and epigenetic levels. Under force stimuli, mechanosensitive ncRNAs with altered expression levels transduce mechanical load to modulate intracellular genes. These ncRNAs regulate the biomechanical responses of periodontium in the catabolic, anabolic, and coupling phases throughout OTM. To achieve this, down or upregulated ncRNAs actively participate in cell proliferation, differentiation, autophagy, inflammatory, immune, and neurovascular responses. This review highlights the regulatory mechanism of fine-tuning ncRNAs in periodontium remodeling during OTM, laying the foundation for safe, precise, and personalized orthodontic treatment.

## Background

Orthodontic tooth movement (OTM) is a complex but highly coordinated biomechanical response to orthodontic forces [1]. During OTM, alveolar bone and the supported periodontal ligament (PDL) react to variations in force magnitude, application time, and directionality through effector cells and signaling cascades [2–4]. The resulting catabolic and anabolic phases require finely tuned modulation; however, the mechanisms have not been fully elucidated. Nowadays, noncoding RNAs (ncRNAs) are receiving increasing interest in the biomechanical and biological processes of OTM [5].

ncRNAs, comprising 98% of total cellular RNA, are classified into two main subgroups: short ncRNAs (< 200 nucleotides) and long ncRNAs (lncRNAs) (> 200 nucleotides) [6, 7]. MicroRNAs (miRNAs) are short ncRNAs of approximately 19–22 nucleotides in length that degrade or suppress mRNAs mainly by pairing with the 3′ untranslated region (UTR) of target mRNAs [8]. The biogenesis of miRNAs begins with pri-miRNAs, which are processed by Drosha into pre-miRNAs and cleaved by Dicer into mature miRNAs [9]. LncRNAs have biogenesis similar to mRNAs and serve important roles in the transcriptional, post-transcriptional, and epigenetic regulation of gene expression as guides, signals, decoys, or scaffolds [10, 11]. For example, lncRNAs contain miRNA response elements to sponge miRNA, thus forming a competing endogenous RNA (ceRNA) to regulate targeted mRNA [12]. ncRNAs are considered significant regulators of cellular functions, biological processes, and several oral diseases, such as periodontitis, cleft lip and palate, and oral cancer [13–18]. Moreover, the expression of ncRNAs alters with dynamic orthodontic mechanical stimuli to orchestrate nuanced remodeling cascades in periodontium [19–21].

To provide the cutting-edge advance of ncRNAs throughout OTM, we summarize the orthodontic force-induced specific expression profiles of ncRNAs and their functions in cellular and molecular responses. Elucidating the delicate regulatory mechanism will provide new clinical practices for safe, precise, and personalized orthodontic treatment.

## Expression profiles of ncRNAs in OTM

**Fig. 1:**
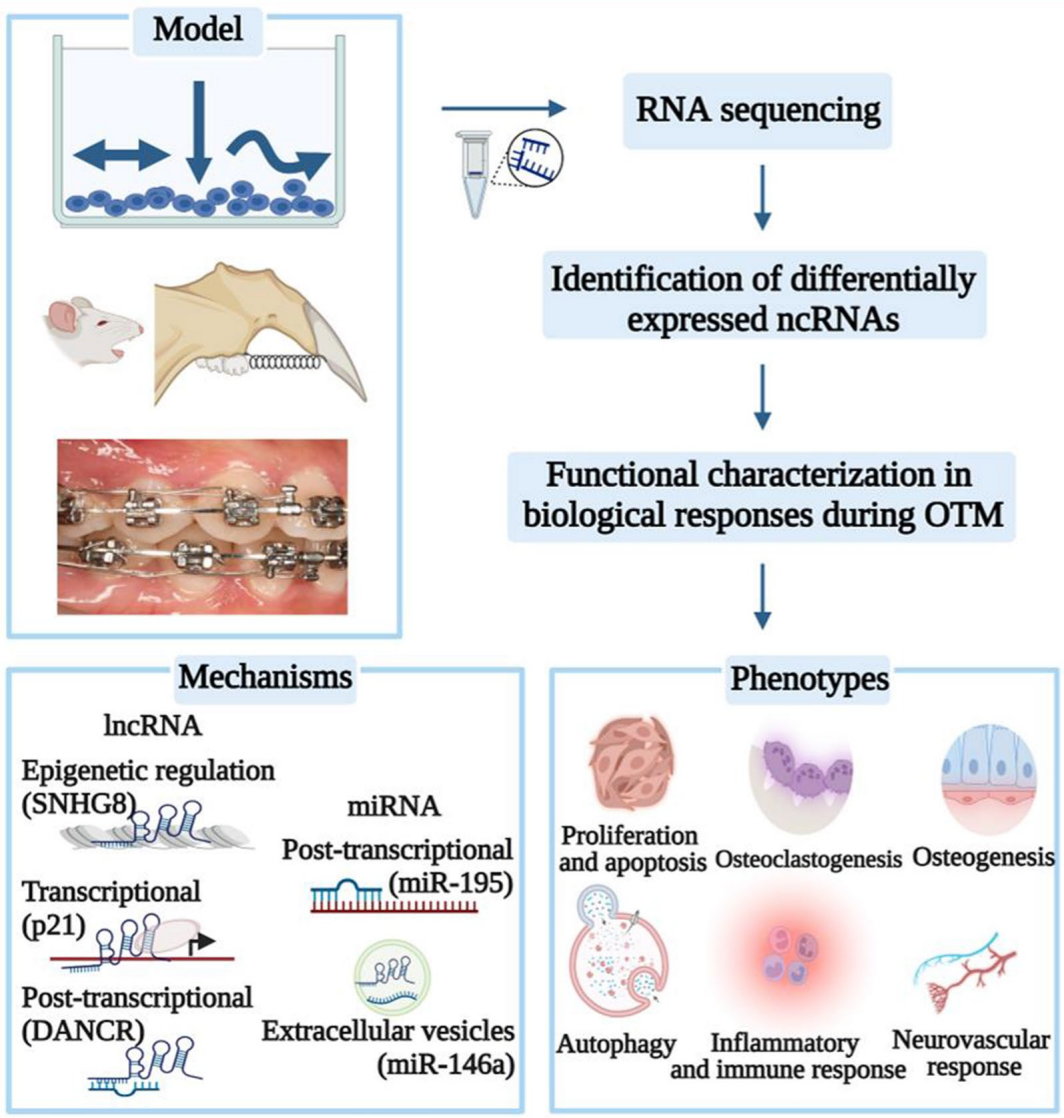
Discovery and characterization of ncRNAs’ functions and mechanisms in OTM. Studies of in vitro force stimulation, mouse or rat OTM models, and samples from orthodontic patients are included in this review. First, comparisons of transcripts from RNA sequencing help identify differentially expressed ncRNAs. Subsequent analyses allow in-depth functional elucidation of ncRNA candidates, which contribute to the biomechanical responses of periodontium by generating phenotypic changes during OTM. Mechanistically, miRNAs regulate gene expression primarily by targeting mRNA, while lncRNAs function at epigenetic, transcriptional, and post-transcriptional levels. Specially, ncRNAs can modulate function of neighbor cells through packaging into extracellular vesicles. In parentheses are some representative examples

Orthodontic loading triggers shear, tension, and compressive deformation of the heterogeneous periodontium. These mechanical signals significantly changed the ncRNA expression profiles in effector cells, including PDL cells (fibroblasts, stem cells, osteoblasts, and osteoclasts) and osteocytes [22]. Other models (i.e., rat, mouse, and human) have also identified some mechanosensitive ncRNAs (Fig. 1). Table 1 displays these ncRNAs and possible interactions to explore the regulatory network in the mechanobiological process of OTM.

**Table 1:** Force-induced ncRNAs and possible interactions in OTM.

Model	Stimulation	Differential expression	Possible Interaction	Study
PDLCs	Stretch force, 12% equibiaxial strain, 0.1 Hz, 12 h	↑: lncRNA CYTOR, MIR22HG, SNHG3	PI3K-Akt, mTOR, Hippo, FoxO, and HIF-1 pathways	[31]
PDLCs, ABCs	Stretch force, 12% deformation, 0.1 Hz, 24 h Compressive force, 2 g/cm^2^, 24 h	**↓**: miR-29 a, b, c ↑: miR-29 a, b, c	ECM genes	[24]
PDLSCs	Stretch force, 10% equibiaxial strain, 1 Hz, 12 h	↑: miR-1246, -5096, -638, -663, -21, -4492, -4734; **↓**: miR-4281, -3178, -3195	MAPK and Wnt signaling pathways	[19]
PDLSCs	Stretch force, 10% equibiaxial strain, 1 Hz, 12 h	**↓**: lncRNA TCONS_00195572; ↑: TCONS_00103186, TCONS_00114231, TCONS_00015104, TCONS_00046925, TCONS_00022234	MAPK and Wnt signaling pathways	[30]
PDLSCs	Stretch force, 10% equibiaxial strain, 1 Hz, 12 h	↑: circRNA3154, circRNA5034, circRNA3133, circRNA5045; **↓**: circRNA1818, circRNA1358	Extracellular vesicular exosome and Wnt signaling pathways	[35]
PDLSCs	Compressive force, 2 g/cm^2^, 12 h	↑: lncRNA FER1L4, HIF1A-AS2, MIAT, NEAT1, ADAMTS9-AS2, LUCAT1; **↓**: MIR31HG, DHFRP1	ECM–receptor interaction, focal adhesion, HIF-1, and PI3K-Akt pathways	[32]
MC3T3-E1 cells	Fluid shear stress, 12 dynes/cm^2^, 1 h	**↓**: miR-20a, -21, -19b, -34a, -34c, -140, -200b;	Osteoblast differentiation	[21]
MC3T3-E1 cells	Stretch force, 2500 µε, 0.5 Hz, 24 h	↑: miR-191, -3070a; ↓: miR-33, -218	Osteoblast differentiation	[99]
OCCM-30	Compressive force, 1.5 g/cm^2^, 8 h	↑: lncRNA Prkcz2, Hklos, Trp53cor1, Gdap 10, Ak312-ps	HIF-1, FoxO, mTOR, Notch, and Rap1 pathways	[148]
C57BL/6 mice	Ni-Ti coil spring, 35 g, 3 d	**↓**: miR-1297, -424-5p, -145-5p, -224-5, -195-5p;	Bone formation	[25]
Wistar rats	Ni-Ti coil spring, 50 g, 3 d	↑: miR-138-5p, -221-3p, -132-3p; **↓**: miR-133a-3p, -133a-5p, -210-3p	Hippo signaling pathway	[147]
PDL tissue from 8 orthodontic patients	Traction interact torsion force, 6 ounces, 4 w	↑: lncRNA DLEU2; **↓**: DNAJC3-AS1	Focal adhesion and PI3K-Akt signaling pathways	[33]
GCF from 15 orthodontic patients	Maxillary canine retraction (T0: pretreatment, T1: 1 h, T2: 1 d, T3: 7 d, T4: 6 w)	↑: miR-29a, b, c (T0-T4); miR-29b: (T0-T1, T0-T3)	Osteoclast activity and periodontal remodeling	[26]
GCF from 20 orthodontic patients	Maxillary canine retraction (T1: 1 h, T2: 1 d, T3: 7 d, T4: 4 w, T5: 12 w)	**↓**: miR-34a (T2–T4)	MMP-2, -9, and -14	[27]

*ABCs* alveolar bone cells; *GCF* gingival crevicular fluid; *MMP* matrix metalloproteinases; *Ni-Ti* nickel-titanium; *OCCM-30* immortalized mouse cementoblast-like cells; *OTM* orthodontic tooth movement; *PDL* periodontal ligament; *PDLCs* periodontal ligament cells; *PDLSCs* periodontal ligament stem cells

### Differentially expressed miRNAs in OTM

The differential miRNA expression profiles could be attributed to types, intensities, and durations of orthodontic forces. The expression of miR-29 a, b, c, and miR-3198 was upregulated under strain force, however, their expression was downregulated under tension [23, 24]. Under the same tension stimulation, a significant decrease in miR-29a, b, c, -193, -101, -27a, b, -33a, -337, and -21 expression was observed at 24 h [24], while a significant decrease in miR-1297, -424-5p, -145-5p, -224-5, and -195-5p was observed after 72 h of stimulation [25]. Gingival crevicular fluid (GCF), derived from orthodontic patients, provides a more accurate overview of the differential miRNA expression profiles. Secretory miR-29 was positively correlated with the duration of force during canine retraction [26]. Unlike miR-29, miR-34a expression was decreased gradually from 1 day to 4 weeks after orthodontic treatment and returned to the pre-treatment level at 12 weeks [27]. These distinct expression patterns suggest that miRNAs mediate different biological responses at different sites and phases of OTM.

A single miRNA could interact with multiple mRNAs and vice versa. For instance, mechanosensitive miR-195-5p directly targeted WNT3A, FGF2, and BMPR1A to affect PDLC osteogenic differentiation under mechanical loading [28]. Efforts are underway to integrate RNA sequencing with in vivo studies to strengthen the OTM process’s miRNA-mRNA network for elucidating the mechanobiological mechanism.

### Differentially expressed lncRNAs in OTM

The investigation into lncRNA-regulated periodontal reconstruction is still in its infancy and provides less extensive information than miRNAs due to the complexity of lncRNAs’ mechanisms of action and the multitude of targets. Tension (10% or 12% equiaxed strain) [29–31], compression force (2 g/cm^2^) [32], and the PDL tissues from orthodontic patients [33] are mainly used to study the differentially expressed lncRNA in OTM. A total of 90 differentially expressed lncRNAs were identified in static stress-treated periodontal ligament stem cells (PDLSCs), while 1339 lncRNAs, including 799 upregulated and 540 downregulated, were identified in response to strain [30]. Under stretching conditions, the lncRNA-mRNA expression profiles in PDLCs have been demonstrated to be highly enriched in PI3K-Akt, an important pathway for osteoblast differentiation [31]. The PI3K-Akt pathway also supported lncRNA-mediated OTM based on the analysis of orthodontic patient data [33].

Circular RNA (circRNA) is a special and relatively stable lncRNA with single-strand covalent closure [34]. 2678 differentially expressed circRNAs were induced in the stretched PDLSCs, and circRNA3140 was suggested to directly or indirectly regulate miRNA-mediated osteogenic differentiation [35]. Specific lncRNAs and circRNAs could function as ceRNAs to promote PDLSC osteogenic differentiation under mechanical force [30, 36, 37]. However, most studies have only performed PCR after RNA sequencing and bioinformatics, without validating the lncRNA-miRNA-mRNA networks.

## Biological roles of ncRNAs in periodontium remodeling during OTM

**Fig. 2:**
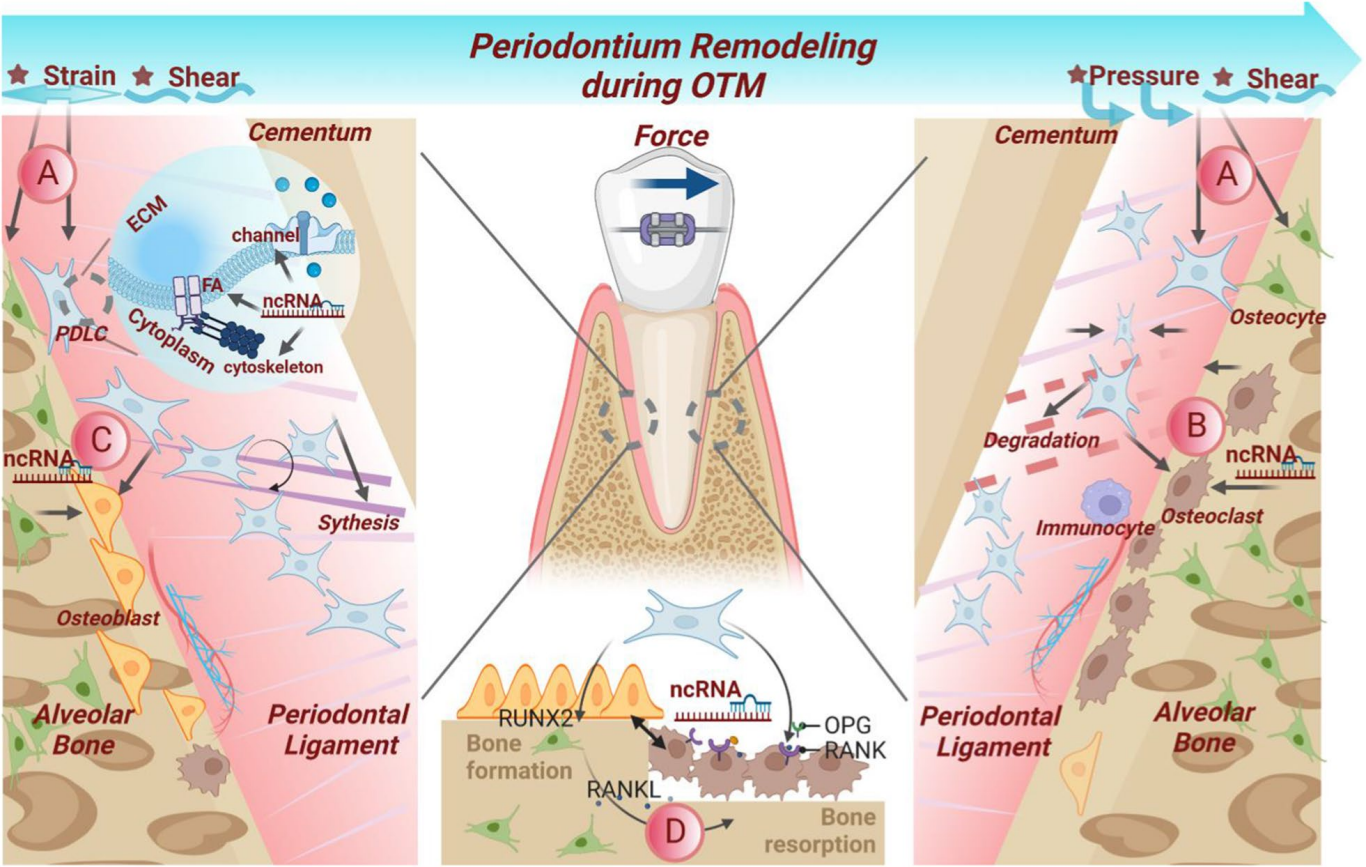
Schematic diagram of ncRNAs regulating periodontium remodeling during OTM. PDL, located between alveolar bone and cementum, is a fibrous connective tissue with neurovascularity and heterogeneous cell groups. **A** ncRNAs contribute to sensing and transducing mechanical forces in osteocytes and PDLCs. **B** In the catabolic phase, ncRNAs are involved in osteoclast-mediated bone resorption and PDLC-mediated PDL degradation. **C** In the subsequent anabolic stage, ncRNAs regulate the function of osteoblasts and PDL synthesis. **D** ncRNAs are responsible for coordinating delicate communication via cytokines or intercellular contact

Orthodontic mechanical signals are initially sensed and transduced by PDLCs and osteocytes [22]. They respond synchronously to stimulate resorption and formation processes in the surrounding periodontium. Based on the recently proposed biphasic theory, the biomechanical responses are histologically divided into catabolic and anabolic phases [38]. ncRNAs are widely involved in the OTM process, from the initial mechanosensing and mechanotransduction to the catabolic and anabolic phases as well as the final periodontium coordination (Fig. 2).

### Mechanosensing and mechanotransduction

Orthodontic force signals are converted and transmitted to nucleus or directly cause nuclear deformation via mechanical sensors, ultimately altering the expression of OTM-related genes and cell behaviors [39]. ncRNAs mediate the OTM mechanosensing and mechanotransduction signals, with a focus on focal adhesion (FA), cytoskeleton, and ion channels as the primary areas of research.

Integrins are crucial mechanoreceptors in OTM and constitute FA together with focal adhesion kinase (FAK), paxillin, and talin. Integrins connect extracellular matrix (ECM) and cytoskeleton with “outside-in” and “inside-out” bidirectional communications [40]. Integrin-mediated FA conformational changes stimulate FAK to transmit mechanical cues via downstream signals, such as the MAPK pathway [41]. miR-138 targeted PTK2, which encodes FAK. By sponging miR-138, lncRNA H19 alleviated PTK2 inhibition, sensitizing mesenchymal stem cells (MSCs) to mechanical tension and inducing osteoblast differentiation [42]. Accordingly, mechanosensitive ncRNAs of increasing importance integrate specific mechanical sensors with extracellular stimuli during OTM.

The cytoskeleton is the hub of mechanical perception and transmission. Orthodontic load acts on cytoskeleton via the FA complex to reshape ECM, thus modulating cellular stress and morphology [43]. Tensile stress activated the RhoA-mediated cytoskeleton assembly in PDLCs [44, 45], whereas compressive stress induced the RhoE-dependent decomposition of stress fiber [46]. ncRNAs interact with Rho/ROCK signaling, the “switches” of actin cytoskeletal structures, to balance the load under fluctuating force. For instance, miR-494-3p targeted ROCK1 to convert the mechanical signal into biological signaling pathways [47]. By directly targeting RhoA, miR-140 suppressed the expression of osteogenesis-related genes and prevented osteogenic differentiation of periodontal ligament fibroblasts [48].

Mechanically-activated ion channels, mainly Piezo1, transduce orthodontic force by triggering calcium influx and the nuclear factor-kappa B (NF-κB) pathway [49]. The differentially expressed miRNAs in mechanically-stimulated PDLSCs enriched calcium transport signaling, contributing to mechanochemical transduction [36]. Downregulation of lncRNA SNHG8 activated the NF-κB signaling pathway during OTM [50]. Such findings suggest that ncRNAs are involved in the expression of genes associated with ion channels, although it is uncertain whether these ncRNAs regulate OTM as an upstream element.

Arguably, cells in PDL and bone are also sensitive to the “passive” mechanical properties of internal adhesion substrates, such as viscoelasticity [51]. PDLCs on a stiffer matrix secreted more ECM proteins like collagen and fibronectin via the mechanical conduction of YAP/TAZ pathway [52, 53]. Mechanosensitive ncRNAs are hypothesized to play a pivotal role in mediating cellular responses to endogenous stress. Three-dimensional culturing of PDLCs in tissue-specific scaffolds is a potential validation direction.

### Catabolic phase

The catabolic phase emphasizes bone resorption and PDL degradation primarily at the pressure zones. Osteoclast-mediated bone resorption occurs after 3 days of force application and lasts 1 to 1.5 weeks [54]. For osteoclast commitment and differentiation, PDLCs, osteocytes, and immune cells jointly upregulated receptor activator of nuclear factor-kappa B ligand (RANKL) and macrophage colony-stimulating factor (M-CSF) by secreting proinflammatory cytokines such as IL-1, IL-6, IL-10, and TNF-α [55, 56]. After differentiation, osteoclasts form ruffled borders, secrete acid into the resorption compartment, and dissolve minerals via cathepsin K (CTSK) and matrix metalloproteinases (MMPs) [57]. Currently, studies on force-induced ncRNAs primarily focus on modulating osteoclastogenic regulatory factors and inflammatory cytokines.

RANKL is a dominant regulator of bone resorption, as proved by blocked OTM in RANKL-depleted PDLCs and osteocytes [58]. RANKL binds to RANK to activate the nuclear factor of activated T cell cytoplasmic 1 (NFATc1) and promote osteoclast fusion and differentiation [59]. Osteoprotegerin (OPG) blocks the signal by acting as a decoy receptor for RANKL. Upregulation of RANKL was observed in PDLCs, osteocytes, and osteoblasts, especially in the first 5 days after application of orthodontic force [60]. In contrast, OPG’s findings seem contradictory [61]. In compressed PDLCs, OPG expression was downregulated, upregulated, or even unchanged, indicating that the expression of osteoclastic regulatory factors exerted a time- and stimulation-dependent pattern [62]. Therefore, the ratio of RANKL/OPG expression, rather than the absolute level of each, determines the osteoclast activity and the resorption pace in each region. miR-21 specifically promoted osteoclast activity with an increased RANKL/OPG level. Conversely, the absence of miR-21 inhibited osteoclastogenesis, as indicated by the reduced OTM distance [63]. Like RANKL, M-CSF is an exogenous osteoclast regulatory factor produced by neighboring cells. M-CSF injection into mice PDL accelerated osteoclast differentiation and subsequently OTM [64]. M-CSF binds to the receptor M-CSFR and promotes the proliferation, adhesion, and migration of osteoclast precursors via the Akt, c-Fos, and ERK signaling pathways [65].

The periodontal attachment apparatus uniquely segregates the mineralized and nonmineralized components. Under orthodontic loading, PDLCs synthesize or degrade ECM depending on various MMPs and tissue inhibitors of metalloproteinases (TIMPs), such as MMP-1, -2, -8 [66–68], TIMP-1, and TIMP-2 [69, 70]. ECM degradation was primarily mediated by increasing MMPs instead of decreasing TIMP levels in compressed PDLCs. The differentially expressed lncRNAs and miRNAs contributed to the decomposition of ECM in compressed PDL [32]. During OTM, the downregulated miR-34 was positively correlated with the expression of MMPs. PDLCs transfected with miR-34a reduced MMP expression and prevented the degradation of ECM [27]. The future challenge is to utilize ncRNAs to control the degradation process and OTM at a steady rate.

### Anabolic phase

The subsequent anabolic phase replenishes the defective bone and PDL, maintaining the position and morphological relationships of new tissue during OTM. Osteoblast precursors and immature osteoblasts originate from MSCs, and further differentiate into mature osteoblasts primarily through Wnt/β-catenin and TGF-β/BMP pathways [71]. Osteoblasts produce and mineralize bone matrix and finally transform into osteocytes in alveolar bone [72]. In the OTM anabolic phase, ncRNAs directly bind to osteogenic transcription factors or indirectly alter the expression of associated molecules to guide osteogenic differentiation and stabilize the cell phenotype.

Osteoblast-mediated bone formation requires at least 3 months to equalize bone resorption, which explains the widening PDL gap after instant OTM. To promote OTM osteogenesis, Wnt/β-catenin signaling is initiated when growth factors bind to cell membrane receptors. Briefly, Wnt1/3a protein binds to the frizzled transmembrane receptor and low-density lipoprotein receptor-related protein 5/6 (LRP5/6), relieves the GSK phosphorylation towards β-catenin, and activates nuclear signaling. Wnt signal was stimulated on the tension side during the 5 days of OTM, while compression decreased the expression of Wnt1 in osteocytes and PDLCs [73]. Notably, knockdown of lncRNA ANCR in PDLSCs activated Wnt/β-catenin and enhanced osteogenic differentiation, while Wnt inhibition blocked this effect [74]. As for TGF-β/BMP signaling pathway, extracellular ligands like TGF-β, BMP, and activin bind to membrane receptors such as ACVR2 to activate R-SMADs, which interact with SMAD4 to induce nucleation and expression of osteogenic genes [75]. Previous studies have shown that several ncRNAs, including miR-106a-5p [76], miR-195-5p [28], and lncRNA MEG3 [77], negatively regulate BMP to inhibit osteogenic differentiation of PDLSCs. By targeting BAMBI and SMAD6, miR-20a enhanced BMP2-mediated osteogenic differentiation under fluid shear stress [78].

Both Wnt/β-catenin and TGF-β/BMP signaling pathways converge and activate transcription factor Runx2. Runx2 binds to the osteoblast-specific cis-acting element 2 (OSE2) and controls the promoter regions of osteogenic genes, including COL I, OPN, OCN, and ALP [79]. A set of upregulated miRNAs (miR-23a, miR-30c, miR-34c, miR-133a, miR-135a, miR-137, miR-204, miR-205, miR-217, and miR-338) inhibited early osteogenic differentiation by directly targeting Runx2 [80]. Conversely, anti-miR-503, anti-miR-103, and anti-miR-195 could upregulate Runx2 to promote osteoblast differentiation in the distraction side of OTM [81].

In this phase, PDLCs produce ECM to coordinate the appropriate PDL width during OTM. The upregulated TIMPs nonspecifically bind to MMPs, thereby inhibiting ECM degradation and promoting its formation. In tensile stimulation studies, the elevated TIMP-1 and TIMP-2 in PDL significantly enhanced the expression of Col1A1, the main ECM component [82, 83]. Upregulated levels of miR-29a, b, and c directly targeted ECM collagen genes to foster ECM during OTM [24]. miR-21 showed high expression levels and decreased in the late stage, which may target PLAP-1 to shape PDL as OTM progresses [84]. Myofibroblasts, newly discovered cells involved in OTM, are highly sensitive to mechanical stimulation and significantly increase collagen production and OCN expression compared to PDLCs [85]. Given that myofibroblasts exert a more favorable effect on the synthesis stage, the regulatory relationship between ncRNAs and myofibroblasts warrants in-depth investigation.

### Coupling between catabolic and anabolic phases

During the turnover process, PDLCs and osteocytes do not govern the differentiation of osteoclasts and osteoblasts separately; instead, they coordinate with the classical RANKL-RANK-OPG signal via autocrine or paracrine cytokines. Moreover, osteoblasts and osteoclasts affect differentiation and function through direct cell-to-cell interactions [86, 87]. Studies have revealed that ncRNA balances the coupling mechanism of periodontium turnover under mechanical loading via cell membrane binding molecules like Notch and Eph/Ephrin and the cytokines like sclerostin.

The Notch signaling pathway includes Notch receptors (Notch1-4) and ligands (Jagged1, 2, and Delta-like1, 3, and 4) on adjacent cell membranes. The activated Notch signal maintains the stemness of MSCs and inhibits osteoclastogenesis and osteoblastogenesis [88]. Studies have shown that miR-34a, b, and c inhibited osteoblast differentiation by targeting Notch1, 2, and Jagged 1 [89, 90]. The expression level of miR-34 was downregulated to positively affect osteogenic differentiation in OTM [27]. Meanwhile, knockdown of lncRNA DANCR reduced osteoclast formation induced by compression force via Jagged1 [91].

Ephrin ligands and receptors are coupling factors that mediate bidirectional communication between osteoblasts and osteoclasts [92]. Stretch-induced PDLCs activated ephrinB2/EphB4 in osteoblasts and stimulated bone apposition [93], whereas compression-induced PDLCs activated ephrin-A2/EphA2, suppressed osteoblast-specific gene expression, and led to bone resorption [94]. ncRNAs are involved in regulating Eph/Ephrin-mediated osteogenesis-osteoclast coordination. For instance, osteoclast-derived miR-214 inhibited osteoblast differentiation via EphinA2/EphA2 interactions, resulting in a sharp decrease in bone formation [95].

Sclerostin, encoded by SOST, stimulates osteocytes to express RANKL and expedites bone resorption during OTM. Meanwhile, sclerostin antagonizes the conduction of Wnt and inhibits new bone formation by binding to LRP5/6 [96]. On the OTM compressed side, sclerostin initially increased and decreased after 5 days. It displayed the opposite pattern on the tension side to offset the negative effect on bone apposition [97]. In the co-culture system, PDLCs upregulated SOST expression in osteocytes via paracrine signaling, thus suppressing bone formation. miR-218 promoted osteogenesis by targeting SOST [98]. Differentially expressed miR-218 was also observed in strain-stimulated osteoblasts and PDLCs [99]. These results suggest that miR-218 may regulate the PDLC-osteocyte-osteoblast cascade via sclerostin, thereby promoting OTM.

These signals tightly control periodontium coupling by gaining osteoblast phenotypes and regulating osteoclast activation. After OTM, the reconstructed PDL and bone are unstable and prone to relapse. This reverse OTM is accompanied by the inward movement of osteoclasts and a delayed response of osteogenic ability. Due to the complex effects on the equilibrium crosstalk between catabolism and anabolism, spatiotemporally expressed ncRNAs could mediate the specific biological effects concerning relapse. Exploiting ncRNAs to regulate these two metabolically opposite but coupled mechanisms seems to be a biological means to assist retention and reduce the risk of recurrence after orthodontic treatment.

## Cellular and molecular mechanisms of ncRNAs in remodeling periodontium

**Fig. 3:**
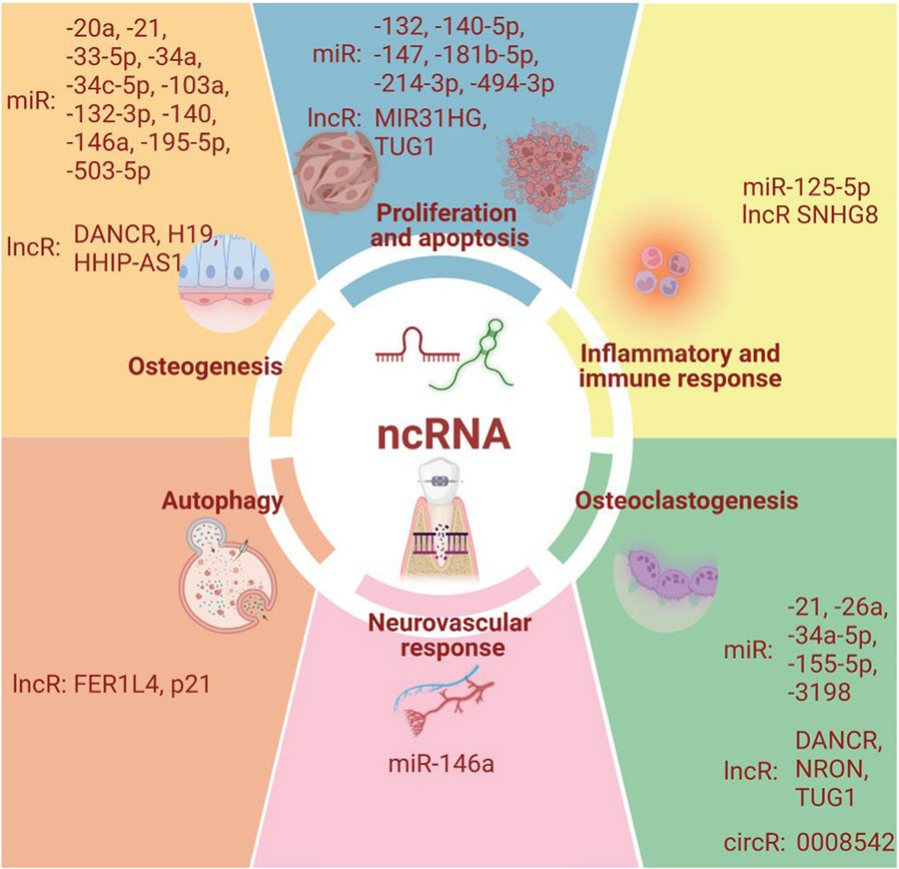
ncRNAs actively participate in cell proliferation, differentiation, autophagy, inflammatory, immune, and neurovascular responses during OTM

Accumulating evidence on the dual roles of ncRNAs underscores the cellular and molecular mechanisms of periodontium remodeling during OTM. ncRNAs function synergistically to regulate cell proliferation, osteoclastogenesis, osteogenesis, autophagy, inflammation, immune, and neurovascular responses (Fig. 3). Differentially expressed miRNAs and lncRNAs exerted enhancing or inhibitory effects by manipulating multiple signaling pathways and cytokines (Tables 2 and 3).

**Table 2:** Effects and mechanisms of miRNAs in periodontium remodeling during OTM.

miRNA	Model	Level	Target	Effect	Mechanism	Study	
miR-20a	MC3T3-E1 cells (fluid shear stress)	**↑**	BAMBI, SMAD	Promoted osteoblast differentiation	BMP2 pathway	[78]	
miR-21	miR-21^−/−^ mice (OTM)	–	PDCD4, RANKL	Promoted osteoclast differentiation	c-Fos/miR-21/PDCD4 positive feedback loop, RANKL/OPG	[110] [63]	
	PDLSCs (stretch force), PDLCs, SD rats (OTM)	**↑**	ACVR2B, PLAP-1, HIF-1α	Promoted osteoblast differentiation	TGF-β pathway	[118] [84, 117]	
miR-26a	PDLCs (compressive force), SD rats (OTM)	**↓**	Promoter targeted by Runx1	Inhibited osteoclast differentiation	Runx1/miR-26a/Jagged1 signaling axis	[115]	
miR-34a	rBMSCs (stretch force), Wistar rats (OTM)	**↑**	GSK-3β	Promoted osteoblast differentiation	Wnt/β-catenin pathway	[124]	
	PDLSCs (stretch force)	**↓**	CELF3	Inhibited osteoblast differentiation	miR-34a/miR146a-CELF3	[120]	
miR-34c-5p	PDLFs (aseptic inflammation)	**↑**	SATB2	Inhibited PDLSC osteogenic differentiation and bone formation	miR-34c-5p/SATB2/ERK	[121]	
miR-125a-5p	PDLCs from orthodontic patients	**↑**	ETV6	Promoted macrophage M2 polarization and osteogenesis	–	[134]	
miR-132	PDLCs (fluid shear stress)	**↑**	–	Promoted proliferation and osteogenic differentiation	PI3K/AKT/mTOR pathway	[102]	
miR-132-3p	MC3T3-E1 cells (stretch force)	**↑**	SMAD5	Inhibited osteoblast differentiation	–	[125]	
miR-140	PDLFs from orthodontic patients	–	RhoA	Inhibited osteoblast differentiation	RhoA-TAZ axis	[48]	
miR-146a-5p	Osteoclasts (compressive force), SD rats (OTM)	**↓**	ADP	Promoted osteoclastogenesis and angiogenesis	miR-146a-ADP feedback loop	[135]	
miR-155-5p	Patients with OIIRR	**↓**	CXCR2	Inhibited osteoclast differentiation	IL-8/CXCR2 axis	[133]	
miR-181b-5p	Osteocyte-like MLO-Y4 cells (stretch force)	**↑**	PTEN	Promoted PDLSC proliferation and osteogenic differentiation	miR-181b-5p/PTEN/AKT signaling	[104]	
miR-195-5p	PDLCs (stretch force), C57BL/6 mice (OTM)	**↓**	WNT3A, FGF2, BMPR1A	Inhibited osteoblast differentiation	WNT and BMP signaling	[28]	
miR-494-3p	MC3T3-E1 cells (compressive force)	**↑**	FGFR2, ROCK1	Inhibited proliferation	–	[47]	
miR-3198	PDLCs (stretch force), (compressive force)	**↓** **↑**	OPG	Regulated osteoclast differentiation	RANKL/OPG	[23]	

*OIIRR* orthodontically induced inflammatory root resorption; *OTM* orthodontic tooth movement; *PDLCs* periodontal ligament cells; *PDLFs* periodontal ligament fibroblasts; *PDLSCs* periodontal ligament stem cells; *rBMSCs* bone stromal stem cells of Wistar rats; *SD* Sprague‑Dawley

**Table 3:** Effects and mechanisms of lncRNAs in periodontium remodeling during OTM.

lncRNA	Model	Level	Target	Effect	Mechanism	Study	
DANCR	MC3T3-E1 cells (compressive force)	↓	–	Reduced DANCR inhibited osteoblast differentiation	NF-κB pathway	[153]	
	PDLCs (compressive force), OCPs	**↑**	Jagged1	Reduced DANCR inhibited osteoclast formation	miR-34a-5p/DANCR-Jagged1	[91]	
FER1L4	PDLSCs (compressive force), BALB/c mice (OTM)	**↑**	–	Promoted autophagy	AKT/FOXO3 pathway	[131]	
H19	BMMSCs (stretch force)	**↑**	miR-138	Promoted osteoblast differentiation	miR-138/PTK2 axis	[42]	
MIR31HG	PDLSCs (compressive force)	**↓**	Prompter targeted by DNMT1 and DNMT3B	Inhibited proliferation	DNMT-mediated DNA methylation	[108]	
Nron	Nron transgenic C57BL/6J mice (OTM)	**↓**	–	Inhibited osteoclast differentiation	Reduced NFATc1 nuclear import	[113]	
p21	IMCC (compressive force), C57BL/6 mice (OTM)	**↑**	FOXO3	Inhibited autophagy and osteoblast function	p21/FOXO3 axis	[130]	
SNHG8	PDLSCs (stretch force), Wistar rats (OTM)	**↓**	Promoter targeted by EZH2	Reduced SNHG8 promoted osteogenic differentiation and bone formation	Erichment of H3K4me3 in the SNHG8 promoter	[123]	
	PDLCs (compressive force), SD rats (OTM)	**↓**	HIF-1α	Reduced SNHG8 promoted hypoxia and inflammation	NF-κB pathway by releasing functional HIF-1α	[50]	
TUG1	MC3T3-E1 cells (fluid shear stress)	**↑**	miR-34a	Promoted proliferation and inhibited apoptosis	TUG1/miR- 34a/FGFR1 axis	[107]	
	PBMCs, SD rats (OTM)	**↑**	MafB	Promoted osteoclast differentiation	–	[114]	
circ_0008542	MC3T3-E1 cells (stretch force)	**↑**	miR-185-5p	Promoted osteoclast differentiation	circ_0008542/miR-185-5p/RANK axis	[111]	

*BMMSCs* bone marrow mesenchymal stem cells; *IMCC* immortalized mouse cementoblast-like cells; *OCPs* human osteoclast precursor cells; *OTM* orthodontic tooth movement; *PBMCs* peripheral blood mononuclear cells; *PDLCs* periodontal ligament cell lines; *PDLSCs* periodontal ligament stem cells; *SD* Sprague‑Dawley

### Regulation of cell proliferation and apoptosis

Coordinated cell proliferation and apoptosis are early OTM steps for tissue homeostasis [100]. Mechanosensitive ncRNAs govern cell fates predominantly at the post-transcriptional level so as to maintain cell numbers and phenotypic plasticity for periodontium remodeling.

In response to fluid shear and tensile forces, ncRNAs expressed at certain levels exert similar proliferative and anti-apoptotic effects via the PI3K/AKT/mTOR and FGF/FGFR signals [101]. The expression levels of miR-132 and cell proliferation rates exhibited similar force-dependent increases after shear stress application. Mechanistically, miR-132 activated the PI3K/AKT/mTOR signaling pathway to promote PDLC proliferation and ECM mineralization [102]. PI3K activates AKT by phosphorylation, promoting the anabolic mTOR complexes and transcription of downstream genes. Specifically, PTEN inhibits the activity of AKT [103]. Under tension stimulation, exosomal miR-181b-5p targeted PTEN to enhance AKT activity and the subsequent PDLSC proliferation [104]. miR-195-5p drove the tension-induced PDLC proliferation and reshaped periodontal tissue by targeting the 3′UTR of FGF2 [28]. Further, shear stress downregulated the expression of miR-140-5p and miR-214-3p, thus stimulating osteoblast proliferation and inhibiting mitochondrial-mediated apoptosis [105, 106]. Unlike the miRNA targeting mRNA mechanism above, lncRNA TUG1 determines cell fate via the well-known ceRNA mechanism. Upregulated TUG1 acted as a sponge to hijack miR-34a, which facilitated FGFR1-mediated cell proliferation and inhibited apoptosis of osteoblasts under shear force [107]. However, the downstream signal of FGF/FGFR remains to be evaluated.

ncRNAs also regulated the proliferation and apoptosis of compression-treated models. Slow growth rate and attenuated cell proliferation were related to several upregulated miRNAs (miR-146a-5p, miR-210-3p, and miR-494-3p) in pressured pre-osteoblasts. Among them, miR-494-3p targeted and downregulated the expression of proliferation-related genes such as FGFR2 and ROCK1 [47]. In another study, downregulated lncRNA MIR31HG inhibited the proliferation of PDLSCs. The force-and time-dependent decrease of MIR31HG was caused by the binding of DNA methyltransferase 1 and 3b to the promoter. That is, CpG island methylation silenced MIR31HG [108].

Notably, cells’ proliferation rate and in vitro lifespan are mainly affected by factors like different donors and cell properties. Therefore, the following aspects, such as the age of orthodontic patients, PDL isolation method, culture medium, and passage time of force effector cells, should be standardized while investigating the role of ncRNA.

### Regulation of osteoclast differentiation and function

**Fig. 4:**
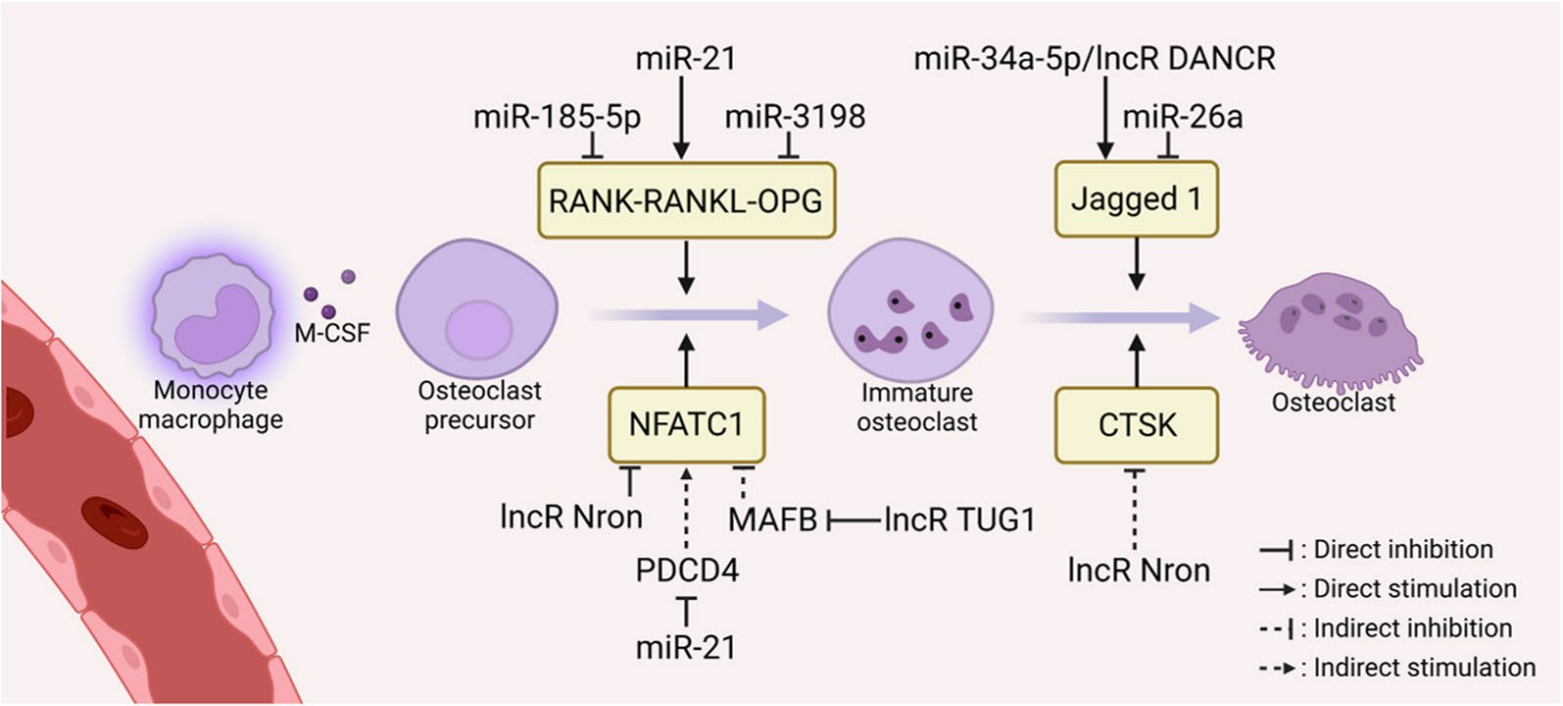
Mechanosensitive ncRNAs regulate the early differentiation of osteoclasts and their functions

Osteoclast activation is the rate-limiting step in OTM. ncRNAs serve as prominent orchestrators in different stages of osteoclastogenesis (Fig. 4). During osteoclast precursor differentiation, some differentially expressed ncRNAs act on cytokine-receptor interactions (RANK-RANKL) and transcription factors (NFATc1), and regulate osteoclast differentiation through cascade signals. During the late stage, some ncRNAs interact with TRAP, CTSK, and Jagged 1 to mediate osteoclast maturation and bone resorption [109].

An interesting facet of ncRNAs on osteoclast differentiation is their strong interrelationship with RANK-RANKL-OPG signaling. miR-21 specifically increased the expression of RANKL, but not OPG or RANK, to optimize osteoclast differentiation during OTM [63]. In a mouse model, miR-21 promoted RANKL-mediated osteoclast differentiation by targeting the 3′UTR of PDCD4. It relieved c-Fos inhibition and led to a temporary reduction in bone mass but an increased OTM [110]. Instead of RANKL, circ_0008542 upregulated RANK by competitively binding with miRNA-185-5p. In tension-induced osteoblasts, circ_0008542 promoted miR-185-5p/RANK-mediated osteoclast differentiation via the m6A methyltransferase METTL3 [111]. In compression-stimulated PDLCs, miR-3198 negatively regulated OPG, enhancing osteoclastic bone resorption. Loss-of-function experiments revealed that OPG was the potential target of miR-3198, not RANKL [23]. Doubtfully, miR-3198 has no orthologue in mice or rats, and further in vivo experiments are needed to verify its role in OTM.

NFATc1 was another bona fide target for differently expressed ncRNAs during OTM [112]. Knockdown of lncRNA Nron in osteoclasts enhanced nuclear translocation of NFATc1, promoted osteoclast differentiation, and expedited alveolar bone resorption and OTM [113]. MAFB negatively regulates NFATc1 and suppresses osteoclastogenesis by interfering with the DNA binding domains of NFATc1, c-Fos, and MITF. During osteoclast differentiation, lncRNA TUG1 expression was elevated and thus precipitated MAFB degradation post-transcriptionally. Overexpression of TUG1 promoted osteoclastogenesis and played a positive role in bone resorption and OTM in vivo [114].

ncRNAs influenced osteoclastic resorption activity via Jagged1, TRAP, and CTSK. Jagged1 and lncRNA DANCR were both dramatically upregulated after compression treatment, and knockdown of DANCR inhibited osteoclast formation via miR-34a-5p/Jagged1 [91]. Additionally, the Runx1/miR-26a/Jagged1 signaling axis was involved in inhibiting osteoclastic response on the pressure side of OTM. Runx1 directly bound to the miR-26a promoter and thereby upregulated its relative expression, which decreased RANKL and numbers of osteoclasts to alleviate Jagged1-mediated bone resorption during OTM [115]. Under orthodontic force loading, TRAP and CTSK reached a peak at 7 days to degrade bone matrix and then decreased in the alveolar bone, whereas lncRNA Nron showed the opposite trend. This phenomenon facilitated osteoclast formation and stimulated bone resorption during OTM [113].

ncRNAs play multi-pronged roles in osteoclast differentiation and collaborate to orchestrate the resorptive activity of the catabolic phase during OTM. Generally, downregulated ncRNAs directly alleviate their anti-osteoclastic effects, whereas upregulated pro-osteoclastic ncRNAs promote osteoclastogenesis. ncRNAs produce the necessity and specificity for osteoclast differentiation and function, which provides a theoretical basis for periodontium metabolism and acceleration of OTM.

### Regulation of osteoblast differentiation and function

**Fig. 5:**
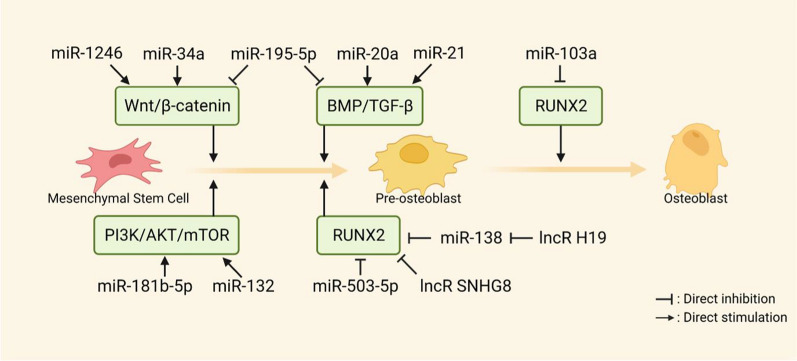
Mechanosensitive ncRNAs regulate osteogenesis through different signals

ncRNAs, as stimulators and suppressors of osteogenic differentiation, play key roles in periodontal tissue modeling under orthodontic load. LncRNAs and miRNAs target or indirectly regulate osteogenic signaling molecules and downstream cascades in an independent or antagonistic manner (Fig. 5).

Several ncRNAs enhance osteoblast differentiation and function under mechanical force by acting on osteogenic factors at the post-transcriptional level. For example, miR-33-5p and miR-20a respectively targeted Hmga2 and SMAD6 to promote tension-induced osteoblast differentiation [78, 116]. Cyclic strain upregulated lncRNA H19 and suppressed miR-138 expression in MSCs, thereby enhancing osteogenic differentiation via the FAK-ERK1/2-Runx2 axis [42]. miR-21 targets multiple genes like HIF-1, PLAP-1, and ACVR2B to promote osteogenic differentiation during OTM. In rat OTM models and hypoxic PDLCs, miR-21 upregulated HIF-1 expression and thus promoted osteogenic differentiation, while the inhibition of miR-21 impeded osteogenic differentiation [117]. A negative correlation between miR-21 expression and PLAP-1 was observed during the PDLC osteogenic differentiation. An increase in miR-21 expression was observed in the early stages of osteoblast differentiation, which decreases during the later stages of bone formation to regulate force-induced periodontal tissue turnover [84]. In stretch-triggered PDLSCs, a time-dependent increase in miR-21 targeted ACVR2B to promote osteogenic differentiation [118]. Based on the lncRNA-miRNA-mRNA network, lncRNA TCONS_00189272 was highly correlated with miR-21 [30], but whether there exists a canonical ceRNA mechanism between them needs to be further investigated.

Unlike the ncRNAs mentioned above, some ncRNAs inhibit osteogenic differentiation and function. For example, miR-503-5p inhibited stretch-induced BMSC osteogenic differentiation and bone formation on the tension side of OTM [119]. Generally, anti-osteogenic ncRNAs interacted directly with the 3**′**UTRs of mRNAs to inhibit the translation of osteogenic-related proteins. miR-146a targeted CELF3 and suppressed osteogenic differentiation of stretched PDLSCs [120]. In the aseptic inflammatory setting of OTM, miR-34c-5p directly bound to SATB2 and inhibited ERK-mediated PDLSC osteogenic differentiation [121]. Another study revealed that miR-195-5p targeted several mRNAs, including WNT3a, FGF2, and BMPR1A, to downregulate osteogenic genes. However, not all direct targets of ncRNA were required for OTM bone formation, such as FGF2 [28]. Mutation in the miR-103a binding site eliminated the Runx2 repression, and miR-103a downregulated Runx2 expression and inhibited tension-triggered osteogenic differentiation [122]. Different from miRNAs, lncRNAs recruited histone modifying enzymes (e.g., EZH2) to the promoter region of Runx2 to induce H3K27me3 methylation. LncRNA SNHG8 exactly repressed Runx2 by this epigenetic modification. SNHG8 expression gradually decreased from 3 to 14 days of OTM, ameliorating the EZH2-mediated Runx2 inhibition and promoting osteogenic differentiation in stretched-PDLSCs [123].

The same mechanosensitive ncRNAs could show seemingly contradictory functions that both supported and inhibited osteoblastogenesis. Under orthodontic force loading, miR-34a promoted osteoblast differentiation in vitro and enhanced local alveolar bone anabolism in vivo [124]. Mechanistically, miR-34a bound to GSK-3β, thereby dephosphorylating β-catenin and initiating the WNT/β-catenin signaling pathway. Despite this osteogenic effect, miR-34a inhibited osteogenic differentiation of stretched-PDLSCs by targeting CELF3 [120]. This contrasting effect on osteoblastogenesis was also observed in miR-132. miR-132 activated the mTOR signaling pathway to promote shear stress-induced PDLSC osteogenic differentiation [102]. However, one isoform of miR-132, miR-132-3p, was upregulated in tension-stimulated pre-osteoblasts and inhibited osteoblast differentiation by targeting SMAD5 of BMP signaling. This suggests that ncRNAs have complex roles in osteoblastogenesis, which might depend on cell types and force parameters [125].

Together, the dual effects of ncRNAs on osteogenic differentiation and function during OTM mainly include the following features: (1) dynamic expression at different stages, such as miR-21; (2) the involvement of feedforward and feedback signals, such as miR-103a and its host genes; (3) coordination with various signaling pathways, such as the action of miR-195-5p on Wnt and BMP. These findings broaden the relationship between ncRNA, mechanics, and bone reconstruction.

### Regulation of autophagy

Autophagy serves as a mechanical adaptation that involves degrading and reusing damaged proteins and organelles in periodontal tissue. It was activated within day 1 of orthodontic loading and was primarily concentrated on the compressed side [126]. The activated autophagic signal exerted complex effects on periodontium remodeling. In PDLCs, autophagy suppressed the osteoclastic response to align PDL [127, 128], while in osteocytes, it promoted RANKL-mediated osteoclast formation [129]. The current study underscores the mechanism of lncRNAs in regulating autophagy under orthodontic stimuli.

During OTM, lncRNAs blocked or activated the autophagic response, primarily by interacting with the autophagic transcription factor FOXO. Nucleus lncRNA p21 directly bound to FOXO3, impeded autophagy and stress-induced cementoblast mineralization at the transcriptional level. In murine OTM models, autophagy activator RAPA partially alleviated p21-mediated cementogenesis inhibition, while autophagy inhibitor 3-MA abrogated the increase in mineralization markers induced by p21 knockdown [130]. LncRNA FER1L4 activated the autophagic cascade by inhibiting phosphorylation of AKT, which triggered nuclear translocation of FOXO3 to minimize periodontal damage on the compressed side of OTM [131]. The complex secondary structure of lncRNA permits interactions with autophagy protein complexes such as FOXO, and whether FER1L4 post-transcriptionally inhibits AKT phosphorylation requires further investigation.

ncRNAs regulate autophagy response to orthodontic loading in a force-dependent and cell-specific manner, which help maximally protect periodontal tissue during OTM. However, autophagy is often accompanied by other stimuli, such as hypoxia and inflammation after orthodontic loading. In vitro and in vivo studies are difficult to interfere with the effects of ncRNAs on autophagy precisely. Further, miRNAs still lack direct evidence for their role in OTM-related autophagy.

### Regulation of inflammatory and immune responses

Sustained orthodontic force stimulated a local aseptic inflammatory response in PDLCs and osteocytes, synergizing with the immune response to promote periodontium reconstruction [132]. Specially, OTM involves the superimposition of existing chronic inflammation with another acute inflammation activated by clinical follow-up. ncRNAs regulate inflammatory and immune responses, which are reflected in inflammatory signaling and immune cells.

Inflammatory molecules such as pro-inflammatory cytokines and chemokines stimulate osteoclast-mediated OTM bone resorption. In compressed PDLSCs, MIR31HG was closely associated with IL-6, which promoted the osteoclastic response on the pressure side of OTM [108]. Fluctuating levels of IL-8 from GCF collection reflected the degree of bone resorption in the corresponding region. CXCR2, a chemokine of IL-8 receptor, was targeted by miR-155-5p to inhibit osteoclast differentiation in the orthodontic inflammatory response [133]. NF-κB signaling pathway modulated the expression levels of inflammatory cytokines. During OTM, downregulated lncRNA SNHG8 released functional HIF-1α to enhance p65 phosphorylation in the NF-κB signaling pathway, thereby promoting periodontal tissue remodeling [50].

Although how immune cells sense mechanical signals is unclear, it is certain that they primarily play a paracrine role in triggering and remitting inflammation during OTM [56]. In miR-21^−/−^ mice, activated T cells promoted RANKL-mediated osteoclast formation, partially attenuating the reduced OTM distance [63]. Pro-inflammatory M1 macrophages primarily activated and stimulated osteoclasts during the early stage of OTM. Conversely, the anti-inflammatory M2 macrophages supported bone deposition. High-level miR-125a-5p in PDL targeted ETV6 to stimulate macrophage M2 polarization, inhibited the NF-κB pathway, and enhanced OTM bone deposition. Further, macrophages exhibited M1 phenotype upon miR-125a-5p suppression [134]. Interestingly, the NF-κB pathway regulates inflammatory and immune responses, and the regulatory functions of ncRNAs give new insights into the acute-to-chronic inflammatory transition process of OTM.

These studies highlight that ncRNAs coordinates with aseptic inflammatory and immune responses to modulate the dominance of inflammatory factors and cellular phenotypes for periodontal tissue metabolism in OTM. It is noteworthy that artificial alteration of OTM by ncRNAs may influence the interconversion and balance of cellular phenotypes and exert adverse effects such as inflammatory root resorption.

### Regulation of neurovascular reaction

The force-induced ischemic and hypoxic periodontium triggers adaptive angiogenesis and neurologic responses during OTM. The neurovascular system provides nutrients and immune cells that help recruit and form osteoblasts and osteoclasts to remodel periodontium.

There is a positive feedback loop between osteoclastogenesis and angiogenesis, and ncRNAs are involved in cell-to-cell communication via extracellular vesicles like exosomes. Compression stress promoted osteoclastogenesis and downregulated exosomal miR-146a in osteoclasts, which targeted adiponectin to promote proliferation, migration, and tubular formation of human umbilical vein endothelial cells (HUVECs). Meanwhile, PDL injected with a miR-146a inhibitor contributed to angiogenesis and the distance of OTM [135]. Osteogenesis has been found to couple with vasculogenesis by secreting vascular endothelial growth factor (VEGF) [136]. Shear force-induced downregulation of miR-140-5p activated the VEGFA/ERK5 signaling pathway and promoted osteoblast proliferation [105]. The intimate crosstalk between angiogenesis, osteoclastogenesis, and osteoblastogenesis regulated by ncRNAs reveals an attractive approach to reconstructing the periodontium.

Nerve fibers not only function as “signal lines” that transduce sensations, such as orthodontic pain, but also as “highways” that transport inflammatory mediators throughout OTM [137]. The activated nerves create a microenvironment that promotes innervated bone regeneration during OTM. In the rat mandibular bone defect model, miR-222 induced BMSC neural differentiation by targeting Nemo-like kinase and supported alveolar bone formation via the Wnt/β-catenin signaling pathway [138]. Unfortunately, few studies have examined the direct effects of orthodontic force-triggered ncRNAs on sensory and sympathetic nervous systems.

Overall, ncRNAs are functionally integrated into the neurovascular responses and exert promotive effects on osteoblasts and osteoclasts in OTM. This represents an emerging field, and future studies are required to focus on the effects and underlying regulatory mechanisms.

## ncRNA-based clinical implications and prospects

**Fig. 6:**
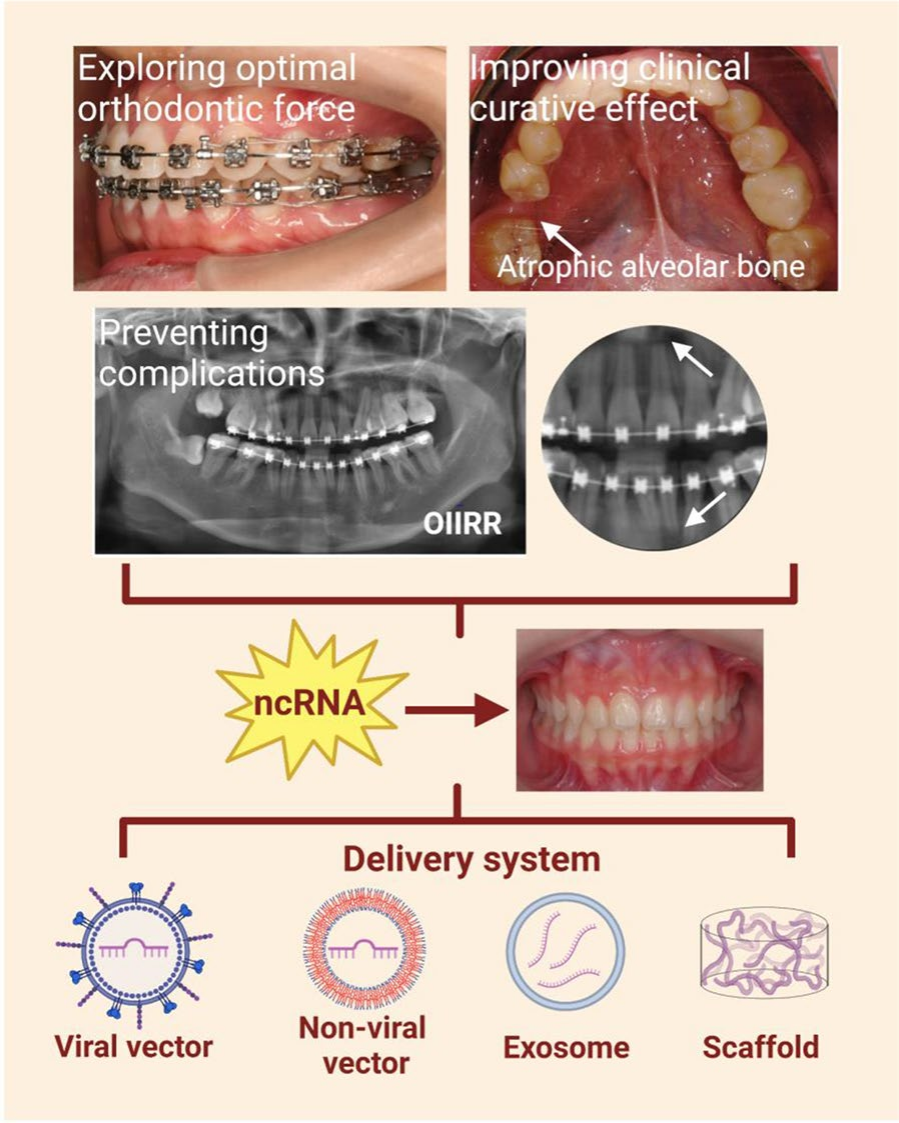
ncRNA-based clinical implications in OTM. Efficient delivery of ncRNAs could be used to explore orthodontic optimal forces, improve clinical outcomes, and prevent complications for achieving desired OTM with minimal side effects

It is a common pursuit of orthodontists to safely, precisely, and efficiently align teeth in the proper three-dimensional position. The interventions of ncRNA on OTM are continually being discovered, promoting the bench-to-clinic translation of personalized orthodontic treatment. The advantages of ncRNA-based therapies are twofold. Firstly, ncRNAs are naturally existing molecules with more precise processing and downstream target selection mechanisms than synthetics [139]. Secondly, ncRNAs act indirectly or directly on genes and signals at multiple levels, eliciting a broad and unique response during periodontium remodeling. In the field of OTM, promising applications of ncRNA include exploring optimal orthodontic force, improving clinical efficacy, and preventing complications (Fig. 6). A summary of carriers on ncRNA therapy is also discussed, aiming at achieving ideal OTM with minimal iatrogenic side effects.

### Exploring optimal orthodontic force

The optimal orthodontic force value is elucidated as the lightest force to achieve the maximum OTM rate; however, its specific value has not come to a conclusion [22]. From a biological perspective, it may be the lightest force that activates osteoclast-mediated tissue resorption. Mechanosensitive ncRNAs have been considered as potential biomarkers during OTM [140]. In GCF collected from orthodontic patients, secretory miR-29 increased immediately after force application and peaked at 4–6 weeks of treatment [26]. Moreover, ncRNA expression profiles at multiple observed times of OTM correlated with osteoclastic function. For instance, a decrease in miR-34a levels was observed after orthodontic treatment and was negatively correlated with MMP expressions [27]. The differently expressed miR-155-5p was involved in different degrees of tissue resorption during OTM [133]. Therefore, optimal forces can be explored by analyzing the time- and force-dependent alterations in ncRNAs’ expression with their functional co-expression profiles.

### Improving clinical curative effect

#### Applications in different orthodontic scenarios

Emerging studies have explored the regulatory mechanism of ncRNAs in several orthodontic clinical scenarios, including functional orthopedic treatment, rapid maxillary expansion (RME), and periodontally accelerated osteogenic orthodontics (PAOO).

Functional orthopedic treatment works with specific muscle forces to move teeth and promote orofacial harmony. While applying functional orthoses, myoblasts undergo proliferation and apoptosis. miR-147 directly bound to the target gene BRSM1, thereby reducing endoplasmic reticulum stress and attenuating cyclic stretch-induced apoptosis in L6 myoblasts [141]. This research presents the potential of targeting ncRNA to adaptively reconstruct the facial muscles in children and teenagers with growth and developmental potential.

RME effectively expands the transverse dimension of the upper arch by opening the midpalatal suture, which is used for treating maxillary hypoplasia. Under RME force, miR-21^−/−^ mice showed impaired cell proliferation, migration, and osteogenic function. RME combined with agomiR-21 (a miR-21 agonist) could be used to accelerate bone formation [142]. The role of miR-21 sheds light on new strategies to control bone turnover, decrease relapse rates, and stabilize the long-term effect of RME.

PAOO accelerates bone metabolism and OTM by performing selective alveolar corticotomy. In the rat model of PAOO, agomiR-21 or antagomiR-21 (a miR-21 antagonist) was injected into the labial, palatal and mesial alveolar mucosa of the first molar prior to surgery. At 7 days post‑PAOO, osteoclast activity and OTM distance were significantly increased following treatment with agomiR‑21, while group antagomiR‑21 displayed the opposite pattern. Mechanistically, miR-21 directly targeted PDCD4 and upregulated c-Fos levels, thereby promoting osteoclastogenesis and loosening the tooth-surrounding alveolar bone [143]. However, the adverse collateral effects, such as cementoclastogenesis and root resorption, pose challenges to future research.

#### Biologically induced orthodontic anchorage

High-angle patients have loose alveolar bone around the supporting teeth and often fail to achieve satisfactory anchorage. On the contrary, low-angle patients are cases with the opposite alveolar bone condition. Local alveolar bone metabolism can be regulated by ncRNAs, which in turn stabilizes or moves the teeth under controlled orthodontic forces.

According to the expression status, ncRNA therapy could be divided into replacement therapy for upregulated expression and inhibition therapy for downregulated expression [144]. For example, miR-503-5p replacement therapy effectively could improve the dense alveolar bone condition in low-angle patients, and mesial tooth movement was achieved for better occlusion and facial profile [119]. LncRNA SNHG8 inhibition therapy unblocked the inhibition of Runx2, promoted local alveolar bone formation, and enhanced anchorage during OTM [123]. The biphasic nature of ncRNA regulation of bone homeostasis makes it a promising direction for biologically induced orthodontic anchorage during OTM. Large-scale data and dose-related studies are still lacking in clinical settings.

#### Complex alveolar bone situation

ncRNAs could accelerate OTM by regulating cell proliferation, differentiation, autophagy, and inflammatory response. However, there is not much room to further speed up the whole OTM process since the reconstruction of periodontal tissue has its own biological rules. Therefore, efficient orthodontic treatment should focus on tooth movement under non-ideal alveolar bone conditions like atrophic alveolar bone, dehiscence, fenestration, and other alveolar bone defects. Besides, osteoporosis or periodontitis can lead to unbalanced bone turnover and uncontrollable OTM, which greatly prolong the treatment course of affected cases.

Several ncRNA therapies are in phase I-III of clinical trials, including miRNA mimics and anti-miRNAs, while no lncRNA-based therapies have entered clinical trials [145]. The treatment of patients with bone metastases and other bone resorption diseases using exogenous miR-34a has entered phase I of clinical trial [146]. During OTM, miR-34a increased osteogenic differentiation and alveolar bone formation by activating the Wnt/β-catenin signaling pathway [124]. miR-503, miR-103, and miR-195 inhibition therapy promoted osteoblast differentiation during OTM by increasing Runx2 expression [81]. Specific ncRNA loading could shorten the clinical course of cases with poor alveolar bone conditions. Interestingly, a targeted upregulation of lncRNA XIST might contribute to safe orthodontic treatment for periodontitis patients [29]. Although ncRNAs improve impaired alveolar bone, the tissue specificity of ncRNA carriers and optimal dose leave room for improvement.

### Preventing complications

Orthodontically induced inflammatory root resorption (OIIRR) is a common complication in orthodontic practice. ncRNAs could be used as biomarkers of OIIRR and help orthodontists detect and reduce OIIRR at an early stage. Studies have shown the involvement of multiple miRNAs, including miR-133a-3p, miR-145-3p, and miR-21-3p, in mechanically induced cementoblast differentiation [147]. Significantly upregulated lncRNAs (Prkcz2, Hklos, Trp53cor1, Gdap10, and Ak312-PS) inhibited cementoblastic function under stress [148]. In orthodontic patients, miR-155-5p inhibited osteoclast differentiation and indicated the degree of OIIRR [133].

As mentioned above, miR-34a-5p/DANCR and Runx1/miR-26a acted together to regulate osteoclast-mediated root resorption via Jagged1 [91, 115]. Additionally, p21 inhibited the autophagy of cementoblasts under stress and impaired cementoblastic functions. The roles of ncRNAs in osteoclastogenesis achieve therapeutic purposes for ameliorating OIIRR. For instance, lentiviral inhibition of p21 rescued impaired cementoblastic differentiation and effectively attenuated OIIRR [130]. However, local injection of lentivirus could lead to off-target inhibition of ncRNAs in PDL resident cells.

### ncRNA delivery systems

Despite the powerful ncRNA intervention on OTM, the specificity and delivery efficiency of ncRNA-based therapies are waiting to be solved due to the degradation and inherent instability of naked ncRNAs. ncRNA delivery systems have been studied in viral, non-viral vectors, exosomes, and scaffolds [144]. Compared to immunogenic viral vectors, non-viral vectors are safer and more modifiable, which are mainly divided into lipid-based and polymer-based vectors. Recently, a polymer-based vector with high transfection efficiency was produced by adding hydrophobic groups onto PEI25K. miR-34a loaded onto this vector notably promoted bone formation during OTM [124]. Unlike other agents, exosomes are natural intercellular nanocarriers of ncRNAs. Studies on exosome-based ncRNA delivery studies have focused on the application of miR-214 in inhibiting bone resorption [95, 149]. Stable exosomes loaded with ncRNAs tend to home to the site of origin when injected into the bloodstream. Thus, exosomes of pro-osteoclastic ncRNAs may preferentially target alveolar bone to accelerate osteoclast-mediated OTM [150]. The use of scaffolds to encapsulate or immobilize ncRNA is an emerging trend. Scaffolds enable three-dimensional distribution and controlled release of ncRNAs in periodontal tissues and can match the transgene expression kinetics of tissue regeneration [151]. A study reported an activated scaffold composed of miR-21 and bio-oss particles, which promoted alveolar bone regeneration in a stable and sustained manner [152]. Despite limited data, ideal functionalized carriers will likely be developed to fine-tune ncRNA for spatiotemporally remodeling periodontium based on mechanical, chemical, and biological innovations.

Taken together, ncRNAs provide new strategies for exploring orthodontic optimal forces, improving clinical outcomes, and preventing root resorption. Specific delivery systems of ncRNA should be further explored to synthesize information about target genes and their pathways for achieving desired tooth movement with minimal side effects.

## Conclusion

ncRNAs display considerable regulatory functions in spatiotemporal periodontium remodeling and hold great promise as biomarkers and therapeutic agents for OTM. In this review, we schematically described the differentially expressed ncRNAs and their complex interaction networks with mRNA to better interpret their roles in OTM. ncRNAs collaborate to regulate biomechanical events during OTM, including mechanosensing, mechanotransduction, catabolism, anabolism, and signal coordination. The intricate regulatory networks of ncRNAs exert coordinated effects on cell proliferation, differentiation, autophagy, inflammatory immune response, and neurovascular activity during OTM. Generally, miRNAs act on target mRNA genes, while lncRNAs regulate cellular functions at the epigenetic, transcriptional, or post-transcriptional level. The multiple sites of action and diverse functions of ncRNA in periodontium remodeling include: (1) dynamic expression at different stages and sites of OTM; (2) involvement in complex feedforward and feedback signals; (3) coordination with various signaling pathways; and (4) mediation of intimate intercellular communication. Identifying and validating ncRNAs and their targets, along with discovering safe and efficient delivery systems, could encourage the transfer of basic research into clinical practice. This review provides an overview of ncRNAs in the underlying mechanisms of OTM and lays the foundation for precise and individualized orthodontic treatment.

## Data Availability

Not applicable.

## Abbreviations

ABC: Alveolar bone cell
ACVR2B: Activin receptor type IIB
ceRNA: Competing endogenous RNA
circRNA: Circular RNA
CTSK: Cathepsin K
ECM: Extracellular matrix protein
FA: Focal adhesion
FAK: Focal adhesion kinase
FGF: Fibroblast growth factor
GCF: Gingival crevicular fluid
HUVEC: Human umbilical vein endothelial cell
lncRNA: Long noncoding RNA
LRP: Low-density lipoprotein receptor-related protein
M-CSF: Macrophage colony-stimulating factor
miRNA: MicroRNA
MMP: Matrix metalloproteinase
MSC: Mesenchymal stem cell
ncRNA: Noncoding RNA
NFATc1: Nuclear factor of activated T cell cytoplasmic 1
OCN: Osteocalcin
OIIRR: Orthodontically induced inflammatory root resorption
OPG: Osteoprotegerin
OSE2: Osteoblast-specific cis-acting element 2
OTM: Orthodontic tooth movement
PAOO: Periodontally accelerated osteogenic orthodontics
PDL: Periodontal ligament
PDLC: Periodontal ligament cell
PDLSC: Periodontal ligament stem cell
PLAP-1: Periodontal ligament-associated protein 1
RANKL: Receptor activator of nuclear factor-kappa B ligand
RME: Rapid maxillary expansion
TIMP: Tissue inhibitors of metalloproteinase
UTR: Untranslated region
VEGF: Vascular endothelial growth factor
